# Paroxysmal Nocturnal Hemoglobinuria in a Young Adult Woman: A Representative Case of Recurrent Intravascular Hemolysis

**DOI:** 10.7759/cureus.100884

**Published:** 2026-01-05

**Authors:** Rita Pera, João Lagarteira, Sara Sá, Rita Diz, Andrei Gradinaru

**Affiliations:** 1 Internal Medicine Department, Unidade Local de Saúde do Nordeste, Bragança, PRT

**Keywords:** anemia, eculizumab, flow cytometry, hemoglobinuria, intravascular hemolysis

## Abstract

Paroxysmal nocturnal hemoglobinuria (PNH) is a rare acquired clonal hematopoietic stem cell disorder characterized by complement-mediated intravascular hemolysis, hemoglobinuria, bone marrow failure, and an increased risk of thrombosis. We report the case of a 39-year-old woman with iron-deficiency anemia unresponsive to oral therapy who presented with three weeks of dark urine and progressive fatigue. Physical examination revealed pallor and mild scleral icterus. Laboratory evaluation demonstrated severe intravascular hemolysis with markedly elevated lactate dehydrogenase, indirect hyperbilirubinemia, undetectable haptoglobin, and a negative direct Coombs test. Urinalysis showed a positive dipstick for blood without erythrocytes on microscopy, consistent with pigmenturia. High-sensitivity flow cytometry confirmed large PNH clones across all cell lineages. Eculizumab therapy was initiated, resulting in the resolution of hemoglobinuria and improvement in hemoglobin levels and symptoms. This case highlights the importance of considering PNH in patients with Coombs-negative hemolytic anemia and recurrent dark urine. Early recognition and timely complement inhibition are essential to reducing hemolysis, preventing thrombosis, and optimizing long-term outcomes.

## Introduction

Paroxysmal nocturnal hemoglobinuria (PNH) is a rare acquired clonal hematopoietic stem cell disorder, with an estimated prevalence of 1-2 cases per million population and an annual incidence of approximately 1.3 cases per million [1,2]. It arises from somatic mutations in the *PIG-A* (phosphatidylinositol glycan anchor biosynthesis, class A) gene, leading to deficiency of glycosylphosphatidylinositol (GPI)-anchored proteins such as CD55 and CD59 on the surface of hematopoietic cells [3]. This defect renders erythrocytes, leukocytes, and platelets highly susceptible to complement-mediated destruction, resulting in intravascular hemolysis, cytopenias, and an increased risk of thrombosis [1,4]. PNH can present at any age but most frequently affects young adults. Its clinical spectrum is highly heterogeneous, ranging from asymptomatic individuals with small PNH clones detected incidentally to patients with overt hemolytic anemia, recurrent hemoglobinuria, bone marrow failure syndromes, and life-threatening thrombotic events involving both typical and atypical vascular sites. Disease severity is influenced by clone size, degree of complement-mediated hemolysis, and the presence of concomitant bone marrow dysfunction [2,3].

Thrombosis, often in atypical sites such as the hepatic, portal, cerebral, and mesenteric veins, remains the leading cause of mortality in PNH [5]. Complement-mediated platelet activation, nitric oxide depletion due to free hemoglobin, and endothelial dysfunction contribute to the prothrombotic state [6]. In addition, patients with PNH may develop a range of chronic complications secondary to sustained intravascular hemolysis and nitric oxide depletion, including progressive renal impairment, pulmonary hypertension, smooth muscle dystonia (manifesting as abdominal pain, dysphagia, or erectile dysfunction), and an increased risk of chronic fatigue and reduced quality of life. These complications contribute significantly to long-term morbidity, even in the absence of overt thrombosis [1,4].

Clinically, patients with PNH may present with fatigue, dyspnea, and recurrent episodes of hemoglobinuria, often more pronounced in the morning, as well as symptoms related to nitric oxide depletion, such as abdominal pain or dysphagia. Laboratory evaluation typically reveals features of intravascular hemolysis, including anemia, markedly elevated lactate dehydrogenase, indirect hyperbilirubinemia, reticulocytosis, and reduced or undetectable haptoglobin, frequently in the setting of a negative direct antiglobulin (Coombs) test [1].

Diagnosis relies primarily on high-sensitivity flow cytometry using fluorescently labeled aerolysin (FLAER) combined with monoclonal antibodies targeting GPI-anchored proteins, including complement regulatory proteins such as CD55 and CD59. This approach enables the detection of even very small PNH clones and helps distinguish PNH from other hemolytic disorders and bone marrow failure syndromes [3].

Recent advances in complement inhibition therapy, most notably with the C5 inhibitors eculizumab and ravulizumab, have transformed the management of PNH by substantially reducing intravascular hemolysis, transfusion burden, and thrombotic risk [4-7]. These agents bind complement component C5 with high affinity, preventing its cleavage into C5a and C5b and thereby inhibiting assembly of the membrane attack complex (C5b-9), which is responsible for complement-mediated erythrocyte lysis [1,3]. Ravulizumab, a long-acting C5 inhibitor, offers similar efficacy to eculizumab but with extended dosing intervals, improving treatment adherence and patient convenience [5,6]. In addition, emerging proximal complement inhibitors, such as danicopan (factor D inhibitor) and pegcetacoplan (C3 inhibitor), target upstream components of the complement cascade, potentially mitigating residual extravascular hemolysis observed in some patients [8,9]. Through effective suppression of terminal or proximal complement activation, these therapies interrupt the central pathophysiological mechanisms of PNH and significantly improve overall survival and quality of life [4-6,8,10]. Early recognition and timely initiation of complement inhibition are essential to modifying disease trajectory and preventing severe complications [1,2,7,10]. Supportive therapies, including red blood cell transfusions, remain important for the management of symptomatic anemia in PNH, particularly during acute hemolytic episodes. In patients with concomitant bone marrow failure syndromes, such as aplastic anemia, immunosuppressive therapy may be indicated to improve hematopoiesis; however, these approaches do not address the underlying complement-mediated hemolysis, which is most effectively targeted by complement inhibition [1,2]. 

We report the case of a 39-year-old woman, previously followed in the immunohematology clinic for iron-deficiency anemia, who presented to the emergency department with dark-colored urine and worsening fatigue over three weeks, illustrating the diagnostic challenges and management considerations in PNH.

## Case presentation

A 39-year-old woman, previously healthy and not on any regular medication, had been followed in the immunohematology clinic for iron-deficiency anemia detected during routine blood tests. She had demonstrated poor response to oral iron therapy and had recently initiated intravenous iron supplementation. Over the preceding three weeks, she experienced recurrent dark-colored urine, particularly in the morning. These episodes were associated with fatigue, mild exertional dyspnea, and occasional dizziness, resulting in multiple emergency department visits. 

On physical examination, she was pale, with mild scleral icterus, but no jaundice of the skin. Vital signs were within normal limits: blood pressure: 118/72 mmHg, heart rate: 92 bpm, respiratory rate: 16/min, oxygen saturation: 98% on room air, and temperature: 36.8°C. No hepatosplenomegaly or lymphadenopathy was noted. Cardiac and respiratory examinations were unremarkable, and neurological evaluation revealed no focal deficits. No petechiae or ecchymoses were observed.

Laboratory evaluation, summarized in Table 1, demonstrated findings consistent with severe hemolytic anemia, including markedly elevated LDH, indirect hyperbilirubinemia, undetectable haptoglobin, and reticulocytosis. Macrocytosis and mild neutropenia were present, with a normal platelet count. Peripheral blood smear revealed a macrocytic anemia with anisocytosis and reticulocytosis, without additional morphological abnormalities. Mild iron deficiency is noted, with normal vitamin B12, folate, and thyroid function. Coagulation studies were within normal limits, and renal function showed a mild increase in creatinine. Inflammatory markers were unremarkable. The direct Coombs test was negative, supporting a non-immune hemolytic process, and complement levels remained within normal ranges. Viral serologies for hepatitis B, hepatitis C, and HIV were negative, and the patient was immune to cytomegalovirus and Epstein-Barr virus.

**Table 1:** Laboratory test results at hospital admission ALP, alkaline phosphatase; ALT, alanine transaminase; APTT, activated partial thromboplastin time; AST, aspartate aminotransferase; CK, creatine kinase; CRP, C-reactive protein; GGT, gamma-glutamyl transferase; INR, international normalized ratio; LDH, lactate dehydrogenase; PT, prothrombin time; TSH, thyroid-stimulating hormone; T4, thyroxine. “-” indicates no normal reference range is applicable.

Parameter	Result	Normal range
Hemoglobin (g/dL)	6.7	12.3-15.3
Erythrocytes (x10^12^/L)	1.80	4.1-5.4
Hematocrit (%)	21.6	35-47
Mean corpuscular volume (fL)	120.0	80-96
Mean corpuscular hemoglobin (pg)	37.2	28-33
Total leucocyte count (x10^9^/L)	4.57	4.4-11.3
Neutrophils (%)	31.7	50-70
Lymphocytes (%)	59.7	25-40
Monocytes (%)	5.5	2-8
Eosinophils (%)	2.0	1-4
Platelet count (x10^9^/L)	243	150-450
Reticulocytes (%)	16.3	0.2-2.0
Haptoglobin (mg/dL)	<10	30-200
Iron (ug/dL)	50	60-180
Ferritin (ng/mL)	150.15	4.63-204.00
Transferrin saturation (%)	22.7	20-50
Total iron-binding capacity (ug/dL)	220.6	215-470
PT (seconds)	11.4	9.4-12.5
INR	1.04	-
APTT (seconds)	30.1	25.1-36.5
D-Dimer (ng/mL)	472	<500
Urea (mg/dL)	33	17-43
Creatinine (mg/dL)	1.2	0.66-1.09
ALT (U/L)	24	<34
AST (U/L)	46	<31
Total bilirubin (mg/dL)	2.55	0.3-1.2
Direct bilirubin (mg/dL)	0.3	<0.2
ALP (U/L)	77	30-120
GGT (U/L)	35	<38
LDH (U/L)	3987	<248
CK (U/L)	128	<145
CRP (mg/dL)	0.08	<0.1
TSH (uUI/mL)	1.93	0.35-4.94
Free T4 (ng/dL)	0.94	0.7-1.48
Vitamin B12 (pg/mL)	487	187-883
Folate (ng/mL)	9.4	3.1-20.5
Immunoglobulin A (g/L)	1.57	0.7-4.0
Immunoglobulin G (g/L)	9.49	7.0-16
Immunoglobulin M (g/L)	1.6	0.4-2.3
Complement C3 (g/L)	0.93	0.9-1.8
Complement C4 (g/L)	0.27	0.1-0.4
Direct antiglobulin test	Negative	-

**Figure 1:**
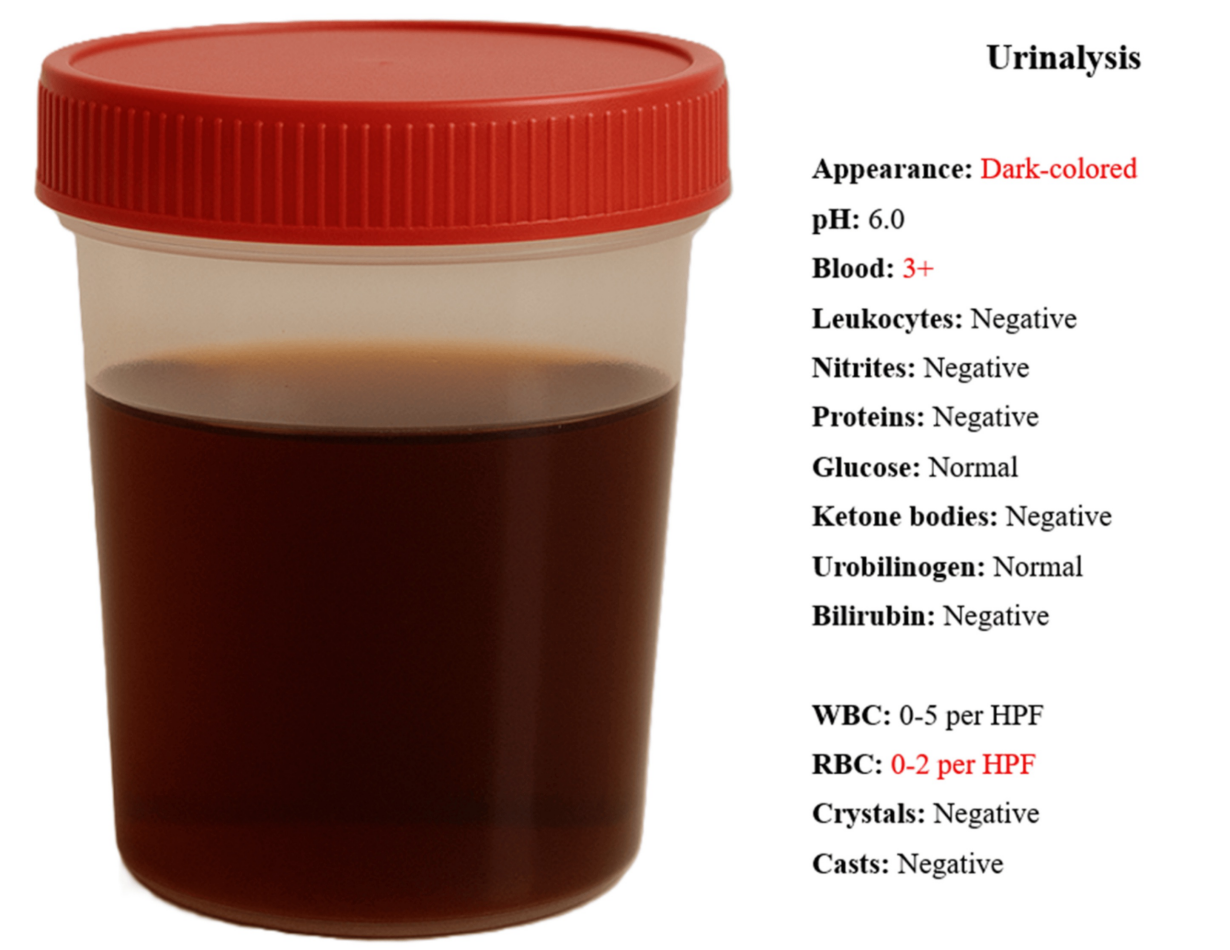
Urinalysis at hospital admission (sample collected at 08:30 AM) HPF, high power field; RBC, red blood cell; WBC, white blood cell.

Urinalysis of an early-morning sample revealed dark-colored urine with a positive dipstick for blood but no erythrocytes on microscopy, consistent with pigmenturia and without evidence of infection or urinary casts (Figure 1). Computed tomography of the abdomen and pelvis showed no abnormalities and no evidence of thrombosis. Upper and lower endoscopic evaluations were unremarkable.

High-sensitivity flow cytometry (lower limit of quantification: 0.01%) identified a large PNH clone across all cell lineages, with GPI-anchor-deficient cells representing 89.59% of neutrophils, 88.30% of monocytes, and 34.60% of erythrocytes (Table 2). These findings support the diagnosis of PNH.

**Table 2:** High-sensitivity flow cytometry results (limit of quantification: 0.01%) showing PNH clone proportions in neutrophils, monocytes, and erythrocytes Cell populations were selected using lineage-specific monoclonal antibodies (neutrophils: anti-CD10; monocytes: anti-CD64; erythrocytes: anti-CD235a/glycophorin A). Detection of GPI-deficient cells was performed using fluorescently labeled aerolysin in combination with monoclonal antibodies targeting GPI-anchored proteins (neutrophils: anti-CD16, anti-CD66b, anti-CD157; monocytes: anti-CD14, anti-CD157; erythrocytes: anti-CD59).

Parameter	Result
Paroxysmal nocturnal hemoglobinuria (PNH) – glycosylphosphatidylinositol (GPI)-anchored erythrocyte proteins	
PNH erythrocytes - CD59-negative (%)	34.600
Normal erythrocytes - Type I (%)	65.400
PNH erythrocytes - Type II, partial deficiency (%)	0.480
PNH erythrocytes - Type III, complete deficiency (%)	34.120
Total PNH erythrocytes (%)	34.600
Paroxysmal nocturnal hemoglobinuria (PNH) – GPI-anchored leucocyte proteins	
Total PNH neutrophil clone (%)	89.590
Total PNH monocyte clone (%)	88.300

Before initiating therapy, the patient received vaccinations against *Streptococcus pneumoniae *(Prevenar 20^®^) and *Neisseria meningitidis* serogroups B and C. Eculizumab was initiated at 600 mg weekly for four weeks, then increased to 900 mg at week five, maintained every two weeks. Following therapy, hemoglobinuria resolved, fatigue improved, and hemoglobin stabilized at 11.2 g/dL by three months. She continues regular hematology follow-up with monitoring of PNH clone size and hemolysis markers, remaining on biweekly eculizumab with sustained clinical and laboratory response.

## Discussion

This case illustrates a classic presentation of PNH, characterized by recurrent episodes of dark urine, severe hemolytic anemia, and marked biochemical evidence of intravascular hemolysis. The patient’s history of “recurrent iron-deficiency anemia” likely reflected chronic hemoglobin and iron loss through hemoglobinuria, a frequent cause of diagnostic delay in PNH [1-3]. The typical morning predominance of hemoglobinuria is explained by nocturnal complement activation and hemoconcentration during sleep, while fatigue relates not only to anemia but also to nitric oxide depletion caused by free hemoglobin, contributing to symptoms such as abdominal pain, dysphagia, and smooth muscle dystonia [1].

Laboratory findings in this case - markedly elevated LDH, indirect hyperbilirubinemia, reticulocytosis, and undetectable haptoglobin - were consistent with uncontrolled intravascular hemolysis [1-3]. Normal C3 and C4 complement levels reinforced that complement activation in PNH is localized to the cell surface rather than systemic [7]. Mild creatinine elevation reflected transient hemoglobin-induced renal stress, a known complication of sustained intravascular hemolysis [4].

High-sensitivity FLAER-based flow cytometry confirmed large GPI-deficient clones, with >80% deficiency in granulocytes and monocytes and 34.6% in erythrocytes, findings strongly associated with disease severity and thrombotic risk [2,3,7]. The discrepancy observed between erythrocyte clone size (34.6%) and the much larger granulocyte and monocyte clones (>80%) is a well-recognized feature in PNH. This difference primarily reflects selective complement-mediated destruction of GPI-deficient erythrocytes during active hemolysis, leading to underrepresentation of PNH red cells in peripheral blood. In addition, recent or prior red blood cell transfusions may further dilute the proportion of PNH erythrocytes, masking the true clone size. In contrast, granulocytes and monocytes are not subject to complement-mediated lysis and therefore more accurately reflect the underlying clonal burden [1,2,7].

Based on the clinical presentation and laboratory findings, this case is most consistent with classical PNH, characterized by predominant intravascular hemolysis and large PNH clones in myeloid lineages. Although mild neutropenia and macrocytosis were present, there was no evidence of pancytopenia or progressive cytopenias suggestive of an underlying bone marrow failure syndrome. Macrocytosis was attributed to reticulocytosis in the setting of active hemolysis, and neutropenia remained mild and stable. Therefore, bone marrow evaluation was considered but deferred, as the overall clinical and laboratory profile did not support PNH associated with aplastic anemia or myelodysplastic features [1].

Thrombosis remains the leading cause of mortality in PNH and frequently involves atypical sites such as the hepatic, mesenteric, or cerebral veins. Thrombotic risk is strongly associated with PNH clone size, particularly when the granulocyte clone exceeds 50% [1,2]. In this case, the presence of a very large granulocyte clone (>80%) placed the patient at increased thrombotic risk. Nevertheless, the absence of clinical, biochemical, and imaging evidence of thrombosis at presentation was notable.

The patient received appropriate pneumococcal and meningococcal vaccination before treatment, reflecting best practice for infection prophylaxis in complement-inhibited individuals [1,7]. Initiation of eculizumab rapidly controlled intravascular hemolysis by blocking terminal complement activation at C5, preventing formation of the membrane attack complex. As expected, a marked improvement in fatigue, resolution of hemoglobinuria, and stabilization of hemoglobin were observed within the first three months of therapy, consistent with published clinical trials and real-world outcomes [4,11,12]. Primary prophylactic anticoagulation was considered; however, it was not initiated, given the absence of prior thrombotic events, normal coagulation parameters, lack of additional prothrombotic factors, and the prompt initiation of complement inhibition therapy, which is known to substantially reduce thrombotic risk [1,4].

Although eculizumab remains a highly effective treatment, its limitations include lifelong biweekly infusions, high cost, and vulnerability to meningococcal infection. Newer complement inhibitors, such as ravulizumab (extended-interval C5 inhibitor), danicopan (factor D inhibitor), and pegcetacoplan (C3 inhibitor), offer alternatives for improved convenience or for patients with residual extravascular hemolysis [5,6,8,9]. Long-term data have shown sustained efficacy, safety, and survival benefits with ravulizumab comparable to or exceeding those of eculizumab [10]. However, as demonstrated in this case, patients with sustained clinical and laboratory remission may continue on eculizumab with excellent outcomes [11,12]. In high-risk patients or those with established thrombosis, anticoagulation remains an important component of management. While vitamin K antagonists have traditionally been used, emerging evidence suggests that direct oral anticoagulants may represent a feasible and safe alternative in selected patients with PNH, although data remain limited and further prospective studies are needed [13].

Regular monitoring of hemolysis markers and PNH clone size remains essential for assessing biochemical response and anticipating breakthrough hemolysis [7]. Given the relatively short follow-up period, ongoing surveillance also includes evaluation for extravascular hemolysis, renal function monitoring, and longitudinal assessment of clone stability to guide future management decisions.

## Conclusions

This case highlights the importance of maintaining a high index of suspicion for PNH in patients with unexplained hemolysis or recurrent dark urine, as delayed recognition remains common and may increase the risk of severe and potentially life-threatening thrombotic complications. High-sensitivity flow cytometry is essential for accurate diagnosis and risk stratification, while prompt initiation of complement inhibition remains critical to halting intravascular hemolysis, preventing thrombosis, and improving quality of life. Although newer complement inhibitors expand therapeutic options, eculizumab continues to provide excellent clinical outcomes in patients with stable disease. Early diagnosis, appropriate vaccination, and close longitudinal monitoring are fundamental components for optimizing long-term management and prognosis in PNH.

## References

[1] Brodsky RA (2014). Paroxysmal nocturnal hemoglobinuria. Blood.

[2] de Latour RP, Mary JY, Salanoubat C (2008). Paroxysmal nocturnal hemoglobinuria: natural history of disease subcategories. Blood.

[3] Richards SJ, Barnett D (2007). The role of flow cytometry in the diagnosis of paroxysmal nocturnal hemoglobinuria in the clinical laboratory. Clin Lab Med.

[4] Hillmen P, Hall C, Marsh JC (2004). Effect of eculizumab on hemolysis and transfusion requirements in patients with paroxysmal nocturnal hemoglobinuria. N Engl J Med.

[5] Dingli D, Messali A, Nag A (2025). Ravulizumab demonstrates real-world effectiveness in patients with paroxysmal nocturnal hemoglobinuria: a US chart review study. Blood.

[6] Lee JW, Sicre de Fontbrune F, Wong Lee Lee L (2019). Ravulizumab (ALXN1210) vs eculizumab in adult patients with PNH naive to complement inhibitors: the 301 study. Blood.

[7] Pu JJ, Brodsky RA (2011). Paroxysmal nocturnal hemoglobinuria from bench to bedside. Clin Transl Sci.

[8] Risitano AM, Kulasekararaj AG, Lee JW (2021). Danicopan: an oral complement factor D inhibitor for paroxysmal nocturnal hemoglobinuria. Haematologica.

[9] Griffin M, Kelly R, Brindel I (2024). Real-world experience of pegcetacoplan in paroxysmal nocturnal hemoglobinuria. Am J Hematol.

[10] Kulasekararaj A, Brodsky R, Schrezenmeier H (2025). Ravulizumab demonstrates long-term efficacy, safety and favorable patient survival in patients with paroxysmal nocturnal hemoglobinuria. Ann Hematol.

[11] Kim JS, Jang JH, Jo DY (2023). Long-term efficacy and safety of eculizumab in patients with paroxysmal nocturnal hemoglobinuria and high disease burden: real-world data from Korea. J Korean Med Sci.

[12] Kelly RJ, Hill A, Arnold LM (2011). Long-term treatment with eculizumab in paroxysmal nocturnal hemoglobinuria: sustained efficacy and improved survival. Blood.

[13] Ali EA, Al-Sadi A, Ali S (2024). Direct oral anticoagulants and paroxysmal nocturnal hemoglobinuria: a systematic review and update on evidence. Cureus.

